# Mobile health lifestyle intervention program leads to clinically significant loss of body weight in patients with NASH

**DOI:** 10.1097/HC9.0000000000000052

**Published:** 2023-03-17

**Authors:** Jonathan G. Stine, Gloriany Rivas, Breianna Hummer, Andres Duarte-Rojo, Christine N May, Nathaniel Geyer, Vernon M. Chinchilli, David E. Conroy, Ellen Siobhan Mitchell, Meaghan McCallum, Andreas Michealides, Kathryn H. Schmitz

**Affiliations:** 1 Division of Gastroenterology and Hepatology, Department of Medicine, The Pennsylvania State University-Milton S. Hershey Medical Center, Hershey, Pennsylvania, USA; 2 Department of Public Health Sciences, The Pennsylvania State University-Milton S. Hershey Medical Center, Hershey, Pennsylvania, USA; 3 Liver Center, The Pennsylvania State University-Milton S. Hershey Medical Center, Hershey, Pennsylvania USA; 4 Cancer Institute, The Pennsylvania State University-Milton S. Hershey Medical Center, Hershey, Pennsylvania, USA; 5 Division of Gastroenterology and Hepatology, Department of Medicine, Northwestern University, Chicago, Illinois, USA; 6 Academic Research, Noom Inc, New York, New York, USA; 7 Department of Kinesiology, The Pennsylvania State University-State College Pennsylvania, USA; 8 Department of Physical Medicine & Rehabilitation, The Pennsylvania State University- Milton S. Hershey Medical Center, Hershey Pennsylvania, USA

## Abstract

Lifestyle intervention remains the foundation of clinical care for patients with NASH; however, most patients are unsuccessful in enacting sustained behavioral change. There remains a clear unmet need to develop lifestyle intervention programs to support weight loss. Mobile health (mHealth) programs offer promise to address this need, yet their efficacy remains unexplored.

**Approach & Results:**

We conducted a 16-week randomized controlled clinical trial involving adults with NASH. Patients were randomly assigned (1:1 ratio) to receive Noom Weight (NW), a mHealth lifestyle intervention program, or standard clinical care. The primary end point was a change in body weight. Secondary end points included feasibility (weekly app engagement), acceptability (>50% approached enrolled), and safety. Of 51 patients approached, 40 (78%) were randomly assigned (20 NW and 20 standard clinical care). NW significantly decreased body weight when compared to standard clinical care (-5.5 kg vs. -0.3 kg, *p* = 0.008; -5.4% vs. -0.4%, *p* = 0.004). More NW subjects achieved a clinically significant weight loss of ≥5% body weight (45% vs. 15%, *p* = 0.038). No adverse events occurred, and the majority (70%) of subjects in the NW arm met the feasibility criteria.

**Conclusions:**

This clinical trial demonstrated that NW is not only feasible, acceptable, and safe but also highly efficacious because this mHealth lifestyle intervention program led to significantly greater body weight loss than standard clinical care. Future large-scale studies are required to validate these findings with more representative samples and to determine if mHealth lifestyle intervention programs can lead to sustained, long-term weight loss in patients with NASH.

## Graphical abstract

**Figure:**
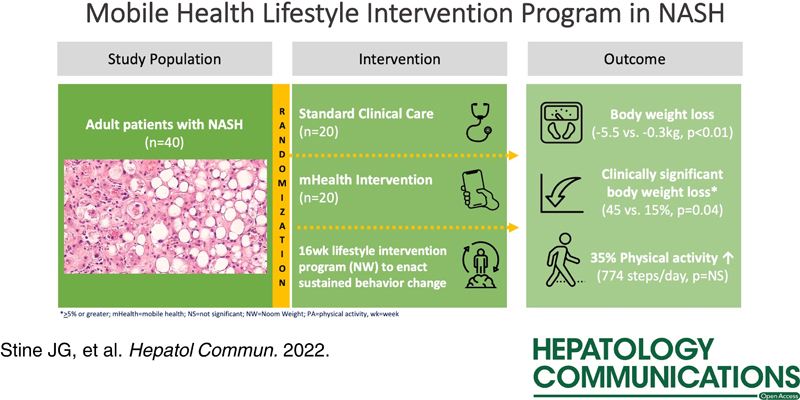

## INTRODUCTION

In the absence of an approved drug therapy or cure, lifestyle intervention with the goal of modest body weight loss continues to be the foundation of clinical management in patients with NAFLD, a leading cause of chronic liver disease worldwide.^1^–^3^ However, most patients are unsuccessful in enacting sustained behavioral change or achieving a clinically significant body weight loss of 5% or greater.^4^,^5^ Self-reported patient barriers to successful lifestyle change include a lack of time, understanding, or access to lifestyle intervention resources.^4^,^5^ Routine lifestyle modification counseling from health care providers continues to be very low in the real-world setting, presenting a further barrier to patients in leading a healthy lifestyle.^6^ Moreover, patients who are overweight or obese experience stigma about their body weight, which negatively impacts their willingness to participate in a lifestyle intervention program centered around body weight loss.^5^,^7^

There is a clear unmet need to develop effective lifestyle intervention programs that can address each of these barriers and empower patients with NAFLD to lead a healthy lifestyle. Such intervention programs need to consider the limitations of patients with NASH, the more severe type of NAFLD. Over the past several years, and forged out of necessity from the COVID-19 pandemic, telehealth and telemedicine have emerged at the forefront of clinical care. Each has been used to help improve the delivery of quality health care to patients with chronic liver disease.^8^,^9^ In patients with NAFLD, intervention with fitness activity trackers^10^,^11^ and directly supervised exercise training programs through real-time secure audio-visual technology^12^ have been successfully utilized in several small pilot studies. While these technologies appear feasible and safe, their impact on clinical outcomes, including body weight, remains unclear as <20% of individuals achieved clinically significant body weight loss.^10^,^12^ Mobile health (mHealth) lifestyle intervention programs, including those that are exclusively smartphone app-based, such as Noom Weight (NW), have been successful in achieving significant loss of body weight in the general population^13^ and in improving clinical outcomes in patients with prediabetes and diabetes.^14^,^15^ Yet, they remain largely unexplored in patients with NAFLD outside of a recent study in Singapore with the Nutritionist Buddy (HeartVoice Pte Ltd) mHealth application.^16^

For these reasons, we conducted a randomized controlled pilot study to determine if a commercially available mHealth-delivered lifestyle intervention program can lead to clinically significant body weight loss in patients with NASH. We also determined the feasibility, acceptability, and safety of this widely accessible application.

## PATIENTS AND METHODS

### Patients and study design

This trial was a 16-week single-center, randomized controlled pilot study (NCT04872777) designed to enroll 40 adult patients with NASH and a smartphone. The study was conducted at Penn State Milton S. Hershey Medical Center and the Penn State College of Medicine in the US. NASH was defined by either (1) a historical liver biopsy with evidence of steatohepatitis [NAFLD Activity Score (NAS) >4]^17^ or (2) an imaging study (eg, ultrasound, CT, or MRI) showing hepatic steatosis and one of the following: (i) Fibrosis-4 (FIB-4) Index ≥1.45 or (ii) vibration-controlled transient elastography (FibroScan, Echosens) with liver stiffness measurement >8.2 kPA or FAST score >0.35.^18^,^19^ Patients were excluded if they were unable to operate a smartphone; participated in a lifestyle intervention program (including a body weight-loss program) within the preceding 90 days; were actively using a body weight-loss supplement; had cirrhosis; had other chronic liver diseases (eg, viral hepatitis); had a secondary cause of hepatic steatosis, including significant alcohol consumption, which was defined as >30 g/d for men and >20 g/d for women; and had severe medical comorbidities or psychiatric illness that would prevent study participation or were unable to provide informed consent.

Patients were randomized 1:1 to intervention with NW, an mHealth-delivered comprehensive lifestyle intervention program that combines smartphone application self-management with human coaching to create and sustain lasting behavioral change (Figure S1, http://links.lww.com/HC9/A184), or standard clinical care using a computer-generated randomization schema (REDCap, Vanderbilt University) in blocks of 10.^20^ Both study groups received standard counseling from an academic hepatologist and in accordance with the best NASH clinical practices, which included Mediterranean-based dietary counseling and a recommendation to complete 150 min/week of moderate-intensity physical activity. Every patient (regardless of study group assignment) was also provided an electronic scale (Fitbit Aria Air, Fitbit Inc, San Francisco, CA), with Bluetooth capabilities that could seamlessly interact with a smartphone (and the NW application for providing feedback on body weight for the NW group). Pertinent clinical data within 28 days before enrollment were captured through a chart review of the electronic medical record where available, and included (1) liver enzymes, (2) blood glucose, (3) hemoglobin A1c, (4) cholesterol, and (5) clinical decision aids [eg, NAFLD Fibrosis Score (NFS) and FIB-4 Index (FIB-4)].

This study was conducted according to the guidelines of the Declaration of Helsinki, Good Clinical Practice guidelines, and local regulatory requirements and was approved by the Penn State College of Medicine Institutional Review Board (No. 17544). It was designed and conducted by the principal investigator in collaboration with the study sub-investigators. Formal written consent was obtained from each participant. The principal investigator collected the data and monitored the conduct of the study. All authors had access to the data, participated in the data interpretation, and can vouch for the accuracy and completeness of the data as well as the fidelity of the trial to the protocol. The final manuscript was reviewed and approved by all authors.

### The Noom Weight application and Noom Coach

NW is an mHealth lifestyle change program that has been shown to promote clinically significant body weight loss and behavior change.^21^ The NW app includes self-monitoring and feedback features for food, exercise, and body weight, as well as digital access to a 1:1 behavior change coach, a support group facilitated by a health coach, and a curriculum delivered through daily articles focused on nutrition, physical activity, and sustainable behavioral change. NW’s approach is informed by cognitive behavioral therapy, acceptance and commitment therapy, and dialectical behavior therapy, all of which aid in behavior change and body weight management.^22^–^24^ Components of these approaches and motivational interviewing are incorporated into the 1:1 coaching and NW’s curriculum. For example, interactive daily articles introduce the framework (eg, What is cognitive behavioral therapy?), describe its components (eg, What are cognitive distortions?), and provide practical tips and applicable examples for the users to incorporate into their life (eg, step-by-step identification and reappraisal of a participant’s cognitive distortion). NW’s curriculum also encourages other dimensions of lifestyle changes that aid in total wellness and weight management by providing relevant lessons regarding other key dimensions of health (eg, paying attention to one’s sleep), physical activity (eg, ideas for integrating exercise into one’s daily life), and stress management (eg, examples of coping skills). In addition to following the curriculum, NW users are also encouraged to log their meals and body weight daily, although the program does allow users who do not wish to weigh in daily to skip that step. Meals are logged according to a tiered system that categorizes foods based on energy density in terms of high, medium, and low energy density. NW users are provided with information on the foods they log, including portion size, calories, caloric density, and other nutritional information. Previous studies have shown that adherence to NW’s color system is associated with greater weight loss over 18 months.^25^ While physical activity counseling is not included in the NW program, the application can access smartphone-based step counts and be used in conjunction with other health and exercise applications (eg, FitBit).

### End points

The primary study end point was a change in body weight as measured by the FitBit Aria Air Bluetooth scale. Secondary end points included feasibility, acceptability, and safety. Across the mHealth literature, there is significant heterogeneity in study design and no agreed-upon standard of feasibility. For these reasons, we defined feasibility according to user engagement.^26^ Following previous studies using NW,^27^ engaged users were defined as those who complete at least 1 meaningful in-application action per week (eg, weight log, food log, exercise log, or article read). Smartphone app utilization data were recorded by Noom’s backend server. Again drawing from our previous experience with lifestyle intervention programs^28^ and from others in patients with chronic liver disease,^29^ acceptability was defined as >50% enrollment of subjects approached. Standard definitions for adverse events, including serious adverse events, were utilized in accordance with local institutional review board policies and definitions, and any known occurrence of serious adverse events in study participants or formal complaints about the intervention were recorded.

Exploratory secondary efficacy end points were calculated by capturing pertinent clinical data within 28 days of study completion where available to include similar data to those that were recorded at the time of study enrollment (listed above).

No significant modifications were made to the original study protocol.

### Statistical power and analysis

Because NW has not been studied in patients with NASH, sample size estimates were derived from previous Noom mHealth interventions in patients with diabetes.^15^ The study sample size was based on the assumption that a 2.5 kg body weight loss would be observed for the lifestyle intervention condition and no change in body weight for the standard clinical care condition (SD of 2.5 kg for each group). With these assumptions and after accounting for 15% subject drop-out^28^,^30^ and a 1:1 enrollment ratio, a total study enrollment size of 40 subjects (20 per condition) would be needed to evaluate the primary end point with 80% statistical power using a 2-sided, 2-sample *t* test and a significance level (alpha) of 0.05.

The analyses included all patients who were randomized. The analyses of the primary end point were performed with the use of both between-group comparisons (2-sample *t* tests) and within-group comparisons (paired *t* tests). Two-sided *p* values of < 0.05 indicated statistical significance, without adjustments for multiple comparisons due to the nature of our study. SAS (Cary, NC) Version 9.4 was used for all statistical analyses.

The secondary clinical end points are presented as means with SD. Both between-group and within-group comparisons were performed. Categorical end points were analyzed with chi-squared and Fisher’s exact test where appropriate. Using similar statistical methods, clinically important subgroup analyses were performed to compare NW responders who lost clinically significant body weight (≥5%) to those who did not.

## RESULTS

### Patient characteristics

**FIGURE 1:**
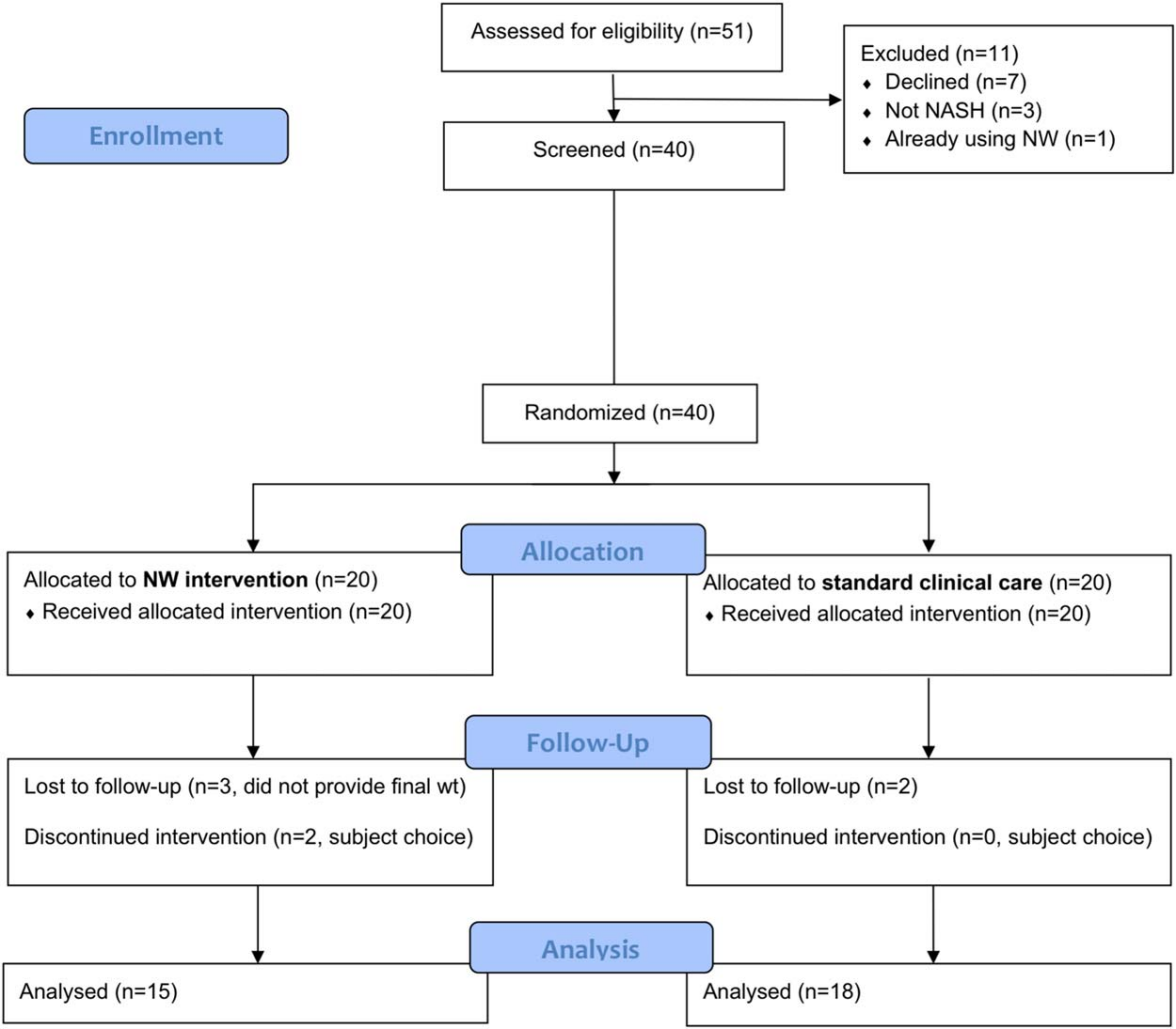
Trial CONSORT Diagram.

From June 2021 through January 2022, 51 patients were approached for this study, 40 (78%) of whom were enrolled (Fig. 1). Twenty patients were randomized and assigned to intervention with NW and 20 patients to standard clinical care. In total, 33 patients (83%) completed the trial (eg, provided a body weight measurement at week 16 through phone call or through self-report in the NW application), with 5 patients in the NW group (1 subject discontinued using NW after wk10 and another subject after wk12, each of whom gained body weight amounts of 3% and 2%, respectively) and 2 patients in the standard clinical care group not completing the trial. Intention-to-treat analysis was performed for all 40 patients assigned to the original groups, where data were available for each of the outcomes of interest.

The mean age of the study participants was 52±13 years (range 24–74 y) and 75% (n=30) were female. The majority of participants were Caucasian (n=38) and 43% (n=17) had a college degree. The mean baseline body mass index (BMI) was 35.5 kg/m^2^ (range 24.7–50.0 kg/m^2^) and the mean baseline body weight was 101.3 kg (range 66.5–148.8 kg). Metabolic comorbidities were common; 63% (n=25) had hypertension, 60% (n=24) had hyperlipidemia, and 45% (n=18) had type 2 diabetes. Mean hemoglobin A1c was 6.8% (range 5.1–8.4%).

For NASH disease staging, 40% (n=16) of the patients had a historical liver biopsy. The other 60% (n=24) had noninvasive disease assessment. In aggregate, individual liver fibrosis stages were as follows: stage F0/F1 (n=17, 43%), stage F2 (n=20, 40%), and stage F3 (n=3, 17%). At baseline, 40% (n=16) of subjects were on stable doses of drug therapies for NASH, including 9 who were taking Vitamin E, 5 receiving a glucagon-like peptide (GLP-1) agonist (semaglutide n=4, liraglutide n=1), and 2 who were prescribed pioglitazone.

There were no significant differences in baseline demographic or clinical characteristics between the NW and standard clinical care groups (Table 1). Specifically, the groups were well matched when comparing body weight, BMI, laboratories, or NASH staging/treatment. Almost all patients (90%) in the NW group were sedentary (<5000 steps/d)^31^ and completed an average of 2239 steps/d the week before beginning the NW program based on smartphone-measured step counts.

**TABLE 1:** Baseline comparisons between Noom Weight and Standard Clinical Care participants

	Noom weight (n=20)	Standard clinical care (n=20)	*p* value
Demographics			
Age, y	53.3 (13.3)	50.4 (12.1)	0.467
Female sex, n (%)	12 (60)	17 (85)	0.077
Race/ethnicity, n (%)			
White	20 (100)	20 (100)	0.487
Hispanic	0 (0)	2 (10)	—
Education, n (%)			
Less than HS	0 (0)	0 (0)	0.613
HS	3 (15)	5 (25)	—
Some college	5 (25)	5 (25)	—
College degree	8 (40)	9 (45)	—
Other/Unknown	4 (20)	1 (5)	—
Smoking status, n (%)			
Active	4 (20)	4 (20)	0.066
Former	1 (5)	6 (30)	—
Never	15 (75)	10 (50)	—
Metabolic risk			
Comorbidities, n (%)			
Diabetes	10 (5)	8 (40)	0.525
Hyperlipidemia	14 (70)	10 (50)	0.197
Hypertension	16 (80)	11 (55)	0.091
PCOS	0 (0)	1 (5)	1.000
Medication use, n (%)			
Cholesterol lowering			
Statin	8 (40)	6 (30)	0.507
Fibrate	2 (10)	0 (0)	0.487
Antihyperglycemic			
Metformin	4 (20)	8 (40)	0.168
GLP-1	3 (15)	2 (10)	1.000
Sulfonylurea	2 (10)	2 (10)	1.000
SGLT2	1 (5)	0 (0)	1.000
Antihypertensive			
ACE/ARB	7 (35)	6 (30)	0.736
Diuretic	5 (25)	3 (15)	0.699
BB	4 (20)	3 (15)	0.677
CCB	1 (5)	2 (10)	1.000
NASH drugs (all)			
Vitamin E	4 (20)	5 (25)	1.000
Pioglitazone	1 (5)	1 (5)	1.000
Blood pressure, mm Hg			
SBP	131 (13.5)	125 (10.9)	0.177
DBP	83 (6.8)	82 (8.3)	0.729
NASH phenotyping			
Fibrosis stage, n (%)			
0/1	8 (40)	9 (45)	0.748
2	10 (50)	10 (50)	—
3	2 (10)	1(5)	—

Note: Randomization worked well as there were no clinically significant differences between the NW group and the standard of care control.

* Continuous variables reported as mean±SD **2-sample *t* tests or chi-squared and Fisher’s exact test.

Abbreviations: ACE indicates angiotensin converting enzyme; ARB, angiotensin receptor blocker; BB, beta blocker; CCB, calcium channel blocker; DBP, diastolic blood pressure; GLP, glucagon-like peptide; HS, high school; PCOS, polycystic ovarian syndrome; SBP, systolic blood pressure; SGLT, sodium-glucose transport protein.

### Efficacy of Noom Weight

**FIGURE 2:**
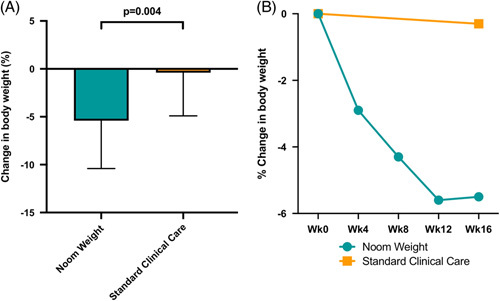
Change in body weight comparing Noom Weight with Standard Clinical Care. (A) After 16 weeks, NW significantly decreased body weight when compared to standard clinical care (-5.4±5.0% vs. -0.4±4.5%, *p* = 0.004). (B) The majority of weight loss was seen by the end of Week 12.

**FIGURE 3:**
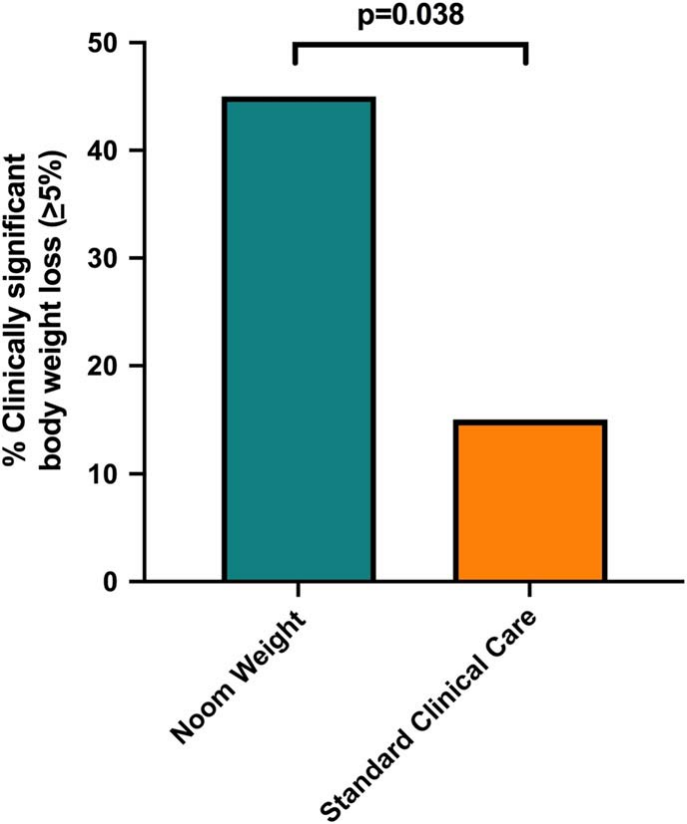
Percent of patients achieving clinically significant body weight loss comparing Noom Weight with Standard Clinical Care. Three times more NW subjects achieved a clinically significant weight loss of >5% baseline body weight compared to subjects receiving standard clinical care (45% vs. 15%, *p* = 0.038).

After 16 weeks, NW significantly decreased body weight when compared to standard clinical care (-5.5±5.8 kg vs. -0.3±4.6 kg, *p* = 0.008; -5.4±5.0% vs. -0.4±4.5%, *p* = 0.004), with the majority of body weight loss observed by the end of week 12 (Fig. 2). More NW subjects achieved a clinically significant weight loss of ≥5% body weight (45% vs. 15%, *p* = 0.038) (Fig. 3). NW also decreased BMI when compared to standard clinical care (-1.5±1.9 kg/m^2^ vs. -0.1±1.6 kg/m^2^, *p* = 0.037; -4.5±5.4% vs. -0.5±4.5%, *p* = 0.031). While not statistically significant, NW marginally increased the baseline physical activity by 35% (774 steps/d measured for a 1-week period at wk 16). No clinically significant changes in readily available clinical outcomes were found, including a change in noninvasive tests, such as liver biochemistries or clinical decision aids (eg, NFS and FIB-4), which were available in up to 40% of subjects (Table 2). There was however a significant reduction in platelet count in the NW group when compared to standard clinical care (-28 vs. -5.7 ×10^9^, *p* = 0.038 (Table 3)). Moreover, no significant between-group differences in baseline characteristics were observed when comparing NW responders who lost clinically significant body weight (≥5%) to those who did not (Table 3).

**TABLE 2:** Outcome measures: noninvasive assessment of liver disease severity and anthropometrics

	Noom weight (n=20)			Standard clinical Care (n=20)			
	Baseline	Post	Within group *p* value	Baseline	Post	Within group *p* value	Between-group *p* value
Clinical Decision Aids							
NFS	−0.52 (1.36)	-0.20 (0.83)	0.444	−0.70 (1.43)	0.85 (1.01)	0.635	0.800
FIB-4	1.39 (1.06)	2.14 (1.53)	0.733	1.67 (1.71)	1.88 (1.61)	0.218	0.322
Anthropometry							
BMI, kg/m^2^	36.1 (6.4)	34.3 (6.5)	0.009	36.3 (6.5)	36.4 (7.1)	0.780	0.037
Weight, kg	105.3 (20.2)	96.8 (19.9)	0.003	97.3 (20.4)	97.7 (22.5)	0.773	0.008
≥5% body weight loss, n(%)	—	9 (45)	—	—	3 (15)	—	0.038

Body weight and BMI were significantly reduced in the NW group. Three times more patients achieved clinically significant body weight loss with NW.

* Both between-group and within-group comparisons were performed. Continuous end points were analyzed with the use of 2-sample and paired *t* tests, respectively, and categorical end points by chi-squared and Fisher’s exact test where appropriate.

Abbreviations: BMI indicates body mass index; FIB-4, Fibrosis-4 index; NFS, NAFLD Fibrosis Score.

**TABLE 3:** Outcome measures: biochemistry

	Noom weight (n=20)			Standard clinical care (n=20)			
	Baseline	Post	Within group *p* value	Baseline	Post	Within-group *p* value	Between-group *p* value
Biochemistry							
Albumin, g/dL	4.5 (0.4)	4.3 (0.2)	0.604	4.5 (0.4)	4.4 (0.4)	0.862	0.753
Alkaline phosphatase, IU/L	86.5 (18.3)	92.0 (15.3)	0.838	77.2 (21.8)	88.4 (30.6)	0.530	0.597
ALT, IU/L	48.6 (27.8)	39.4 (17.4)	0.547	51.5 (29.7)	46.3 (11.8)	0.269	0.644
AST, IU/L	41.2 (19.1)	36.9 (16.3)	0.310	38.0 (23.0)	34.1 (11.3)	0.250	0.602
Glucose (fasting), mg/dL	125.0 (42.7)	117.1 (12.3)	0.609	127.5 (56.9)	153.1 (68.7)	0.143	0.249
Platelet count, 10^9^	247.0 (60.5)	219.0 (27.1)	0.109	235.7 (105.7)	230.0 (130.3)	0.200	0.038

A reduction in platelet count was observed with NW, otherwise, no statistically significant changes were observed in the laboratories obtained through chart review.

* Both between-group and within-group comparisons were performed. Continuous end points were analyzed with the use of 2-sample *t* tests and paired *t* tests

** reported as mean±SD

ALT indicates alanine aminotransferase; AST, aspartate aminotransferase; DBP, diastolic blood pressure; HDL, high density lipoprotein; HOMA-IR, homeostatic model assessment of insulin resistance; LDL, low density lipoprotein; SBP, systolic blood pressure; WBC, white blood cell.

### Noom Weight utilization and engagement

Overall utilization of the NW application was quite high; 83% of the available weeks had measurable subject interaction with the smartphone application. Over time, NW opening rates each week remained relatively constant until week 16 (Figure S2, http://links.lww.com/HC9/A185), where there was a 15% decline in app opening. Peak NW utilization and engagement occurred at week 4, which coincided with the greatest rate of body weight change (Fig. 2 & S2, http://links.lww.com/HC9/A185). Average weekly NW engagement per subject was as follows for self-directed activities: 17 meal logs; 15 content reviews; 4 weigh-ins; 2 exercises, and 1 coach message. Self-directed activity choice changed weekly (Figure S3, http://links.lww.com/HC9/A186).

The social component of the application was utilized much less frequently. Average NW engagement per subject for social activities included interaction with 1 coaching message and 1 group post/comment/like.

**FIGURE 4:**
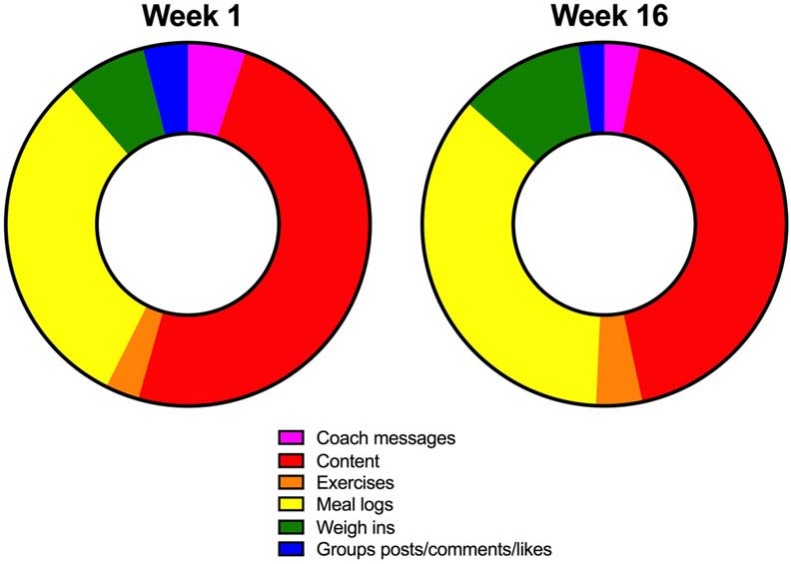
Temporal changes in Noom Weight engagement. Over time, engagement with the Noom Weight app became more self-directed, and participants spent a greater percentage of their time with meal logging (yellow) or weigh-ins (green).

A behavioral shift was observed between week 1 and week 16. Over time, NW participant engagement became more self-initiated, with a greater percentage of application engagement spent with meal logging (29.2% week 1 vs. 49.0% week 16, *p* = 0.024) or weigh-ins (7.1% week 1 vs. 15.1% week 16, *p* = 0.180) rather than interactions with the Noom coach (4.4% week 1 vs. 1.7% week 16, *p* = 0.064) or educational content (54.5% week 1 vs. 32.9% week 16, *p* = 0.016) (Fig. 4).

### Feasibility, acceptability, and safety of Noom Weight

The majority (70%) of subjects met the *a priori* definition of feasibility by interacting with the NW smartphone application at least once each week. The *a priori* definition of acceptability was also met because 78% of the patients approached decided to enroll in this clinical trial. Importantly, no adverse events were reported.

## DISCUSSION

This is the first study to examine an mHealth-delivered lifestyle intervention program in adults with NASH. Among patients with NASH, 16 weeks of NW was superior to standard clinical care with respect to body weight loss because patients who completed NW lost 5 times more body weight overall, with nearly half of the NW patients achieving clinically significant body weight loss of 5% or greater. This was seen independent of baseline body weight, BMI, individual components of the metabolic syndrome (eg, diabetes and hypertension), or NASH severity. Moreover, the NW lifestyle intervention program is feasible, acceptable, and safe as the *a priori* definition of each was met at the conclusion of this pilot clinical trial. Taken together, these data provide novel information that NW is not only feasible, acceptable, and safe but also highly efficacious in that this mHealth-delivered lifestyle intervention program led to significantly greater body weight loss than standard clinical care.

Because at this time there is no known cure or regulatory agency-approved drug therapy for patients with NASH, an effective lifestyle intervention program will be crucial in improving patient-oriented outcomes, including the reduction of major adverse liver outcomes, major adverse cardiovascular events, and primary liver as well as extrahepatic cancers. Clinical guidelines from multiple leading academic societies^1^,^32^ focus broadly on lifestyle modification with dietary change and increased physical activity for all patients with NAFLD, including those with NASH; yet, they do not specify how such change is to be carried forth. Our findings suggest that mHealth is an effective platform by which to deliver a lifestyle intervention program and that the combination of smartphone application self-management with human coaching created and sustained lasting behavioral change through the NW program in patients with NASH.

Based on the findings of Vilar Gomez et al,^33^ current clinical guidelines suggest a body weight loss of at least 5% to improve not only liver fat but also the histologic features of NASH. Importantly, 45% of patients who participated in the NW program achieved 5% body weight loss or greater. This rate is much greater than that of the 30% of patients with NASH who achieved this threshold of weight loss published by Vilar Gomez et al,^33^ who received in-person instruction to consume a hypocaloric diet and complete at least 200 min/week of habitual physical activity followed by individual in-person instruction every 8 weeks. Also, while this pilot study did not directly compare a supervised in-person lifestyle intervention program, such as the one used by Vilar Gomez et al.,^33^ it is nonetheless intriguing and promising to see this indirect comparison in which NW outperforms historical intervention data in achieving rates of clinically significant body weight loss. Moreover, NW appears to be at least as effective in body weight loss when compared to other less widely available mHealth apps that have reported 25% of subjects achieving a clinically significant weight loss.^15^

We also found a shift in behavior with the NW smartphone application. Over the duration of the 16-week program, participants initiated more interactions with the NW mobile application and spent a greater percentage of their time engaged with the application in self-monitoring and feedback activities such as meal logging or weigh-ins. This is important because self-monitoring has long been thought to be a central component of the behavioral treatment for weight loss,^34^ including successful long-term weight management.^35^ These data suggests that over time, NW may create the self-regulatory skills and feedback required for long-term success and create an intriguing avenue for follow-up study.

Our study has multiple strengths inherent to the study design (eg, in the use of a commercial mHealth smartphone app-delivered lifestyle intervention program that incorporates human coaching, NW is efficacious in other populations with metabolic disease), study population (eg, a well phenotyped cohort of patients with NASH), possibly cost (eg, a monthly NW subscription may be less costly than a fitness center membership, personal training, or dietary counseling with a Registered Dietitian),^36^ and convenience factor as smartphone-based apps allow for program access from anywhere and are not tied to a physical location, such as a gym or wellness center. Limitations include the sample size, a largely Caucasian and female population, which may limit the generalizability, lack of allocation concealment, inability to capture NASH-relevant clinical end points in each patient (this was a pilot study), lack of histologic outcome (body weight reduction was used as a surrogate for histologic response), and no long-term follow-up. We also excluded patients with cirrhosis, including those without clinically significant portal hypertension, where body weight loss may still be helpful, offering an important avenue for future study.

Future studies with large sample sizes, blinded assessors, and longer follow-up are necessary to confirm the results of this pilot study and to ascertain the sustainability of the findings. However, the present study demonstrates that clinically significant body weight loss is possible with only a short-term mHealth smartphone app-delivered lifestyle intervention. This finding is meaningful because it provides the basis for future large-scale use of smartphone-based lifestyle intervention for the clinical management of patients with NASH.

## CONCLUSION

This randomized controlled clinical trial demonstrated that NW is not only feasible, acceptable, and safe but also highly efficacious in that this mHealth-delivered lifestyle intervention program led to significantly greater body weight loss than standard clinical care and at rates much greater than previous mHealth-based lifestyle intervention programs. Moreover, in a short time period, NW led to the establishment of self-monitoring behaviors necessary for long-term weight management success. Future large-scale studies with long-term follow-up are required to determine if mHealth-delivered lifestyle intervention programs can lead to sustained, long-term weight loss and improvement in routine clinical outcomes in patients with NAFLD and NASH.

## Supplementary Material
