# Supplementary figures and images for: Mobile health lifestyle intervention program leads to clinically significant loss of body weight in patients with NASH
Source: Hepatol Commun. 2023 Mar 17;7(4):e0052. doi: 10.1097/HC9.0000000000000052 (PMC10027041; doi:10.1097/HC9.0000000000000052)

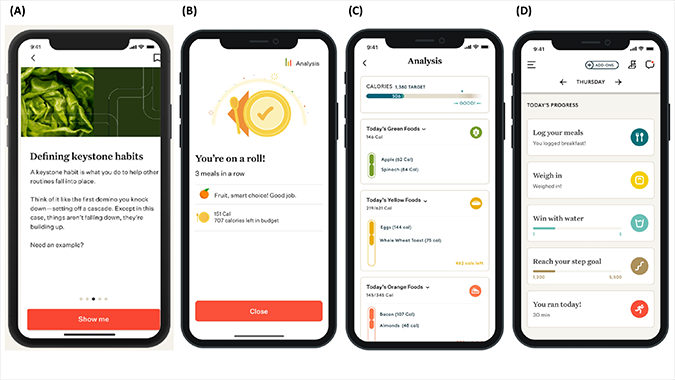

Supplement: Supplementary file 1 [file hc9-7-e0052-s001.tiff]

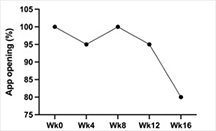

Supplement: Supplementary file 2 [file hc9-7-e0052-s002.tiff]

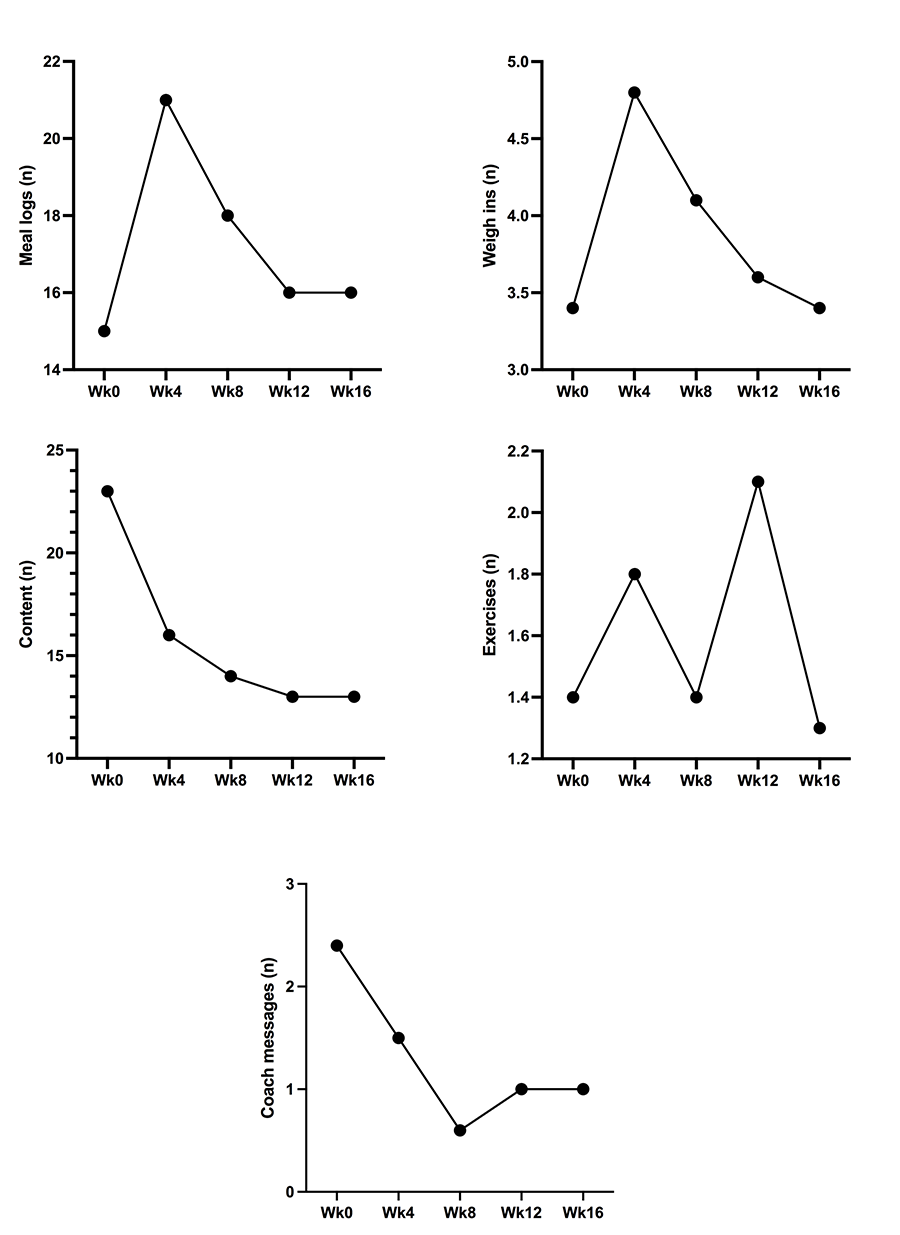

Supplement: Supplementary file 3 [file hc9-7-e0052-s003.tiff]
